# Concomitant plasmacytoma and B cell lymphoma with discordant light chain expression but clonal identity

**DOI:** 10.1007/s00277-012-1443-0

**Published:** 2012-03-08

**Authors:** Wing Y. Au, Lau Wai-Hung, Kai Y. Wong, William W. L. Choi

**Affiliations:** 1 Department of Medicine, Queen Mary Hospital, The University of Hong Kong, UMU, 4/F, Professorial Block Pokfulam Road, Pok Fu Lam, Hong Kong; 2 Department of Pathology, Queen Mary Hospital, The University of Hong Kong, Pok Fu Lam, Hong Kong

**Fig. 1:**
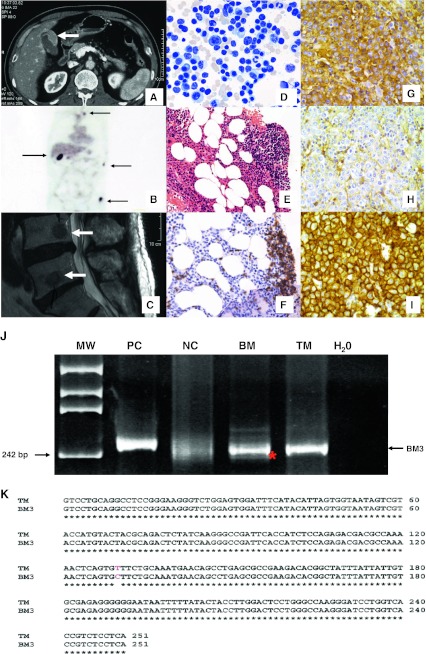
**a** Computerized tomogram scan showing a gallbladder lesion (*arrow*). **b** Positron emission tomogram scan showing FDG avid uptake (*arrows*) in the porta hepatis as well as in the sternum, ribs, and femur. **c** Magnetic resonance imaging showing osteolytic lesions in multiple vertebra levels. **d–f** Marrow aspirate and trephine biopsy showing heavy lymphoma infiltration with no evidence of plasma cell dyscrasia. The B cells were highlighted by CD20 staining (**f**). **g–i** The gallbladder lesion showed sheets of plasma cells with CD138 expression (**g**) negative for kappa light chain (**h**) but expressing lambda light chain (**i**). **j** Polymerase chain reaction using IgH primers on DNA extracted from marrow and gallbladder lesions showing bands of similar band size (*asterisk* and *arrow*). *MW* molecular weight control, *PC* positive control, *NC* negative control, *BM* bone marrow, *TM* tissue material from gallbladder, *H*_*2*_ *O* water blank. **k** Cloning, sequencing, and alignment showing homology between clones from the two discordant lesions

Dear Editor,

A 63-year-old patient presented with abdominal pain. A computerized tomogram scan showed a gallbladder mass (Fig. 1a). A positron emission tomogram revealed additional FDG-avid lesions in the sternum, ribs, bilateral humeri, vertebral column, and pelvic bone (Fig. 1b). Magnetic resonance imaging confirmed vertebral lytic lesions (Fig. 1c). A serum protein electrophoresis showed a monoclonal band (IgA λ 16.2g/l), and he was referred for suspected plasma cell myeloma (PCM). A bone marrow biopsy, however, showed 19 % infiltration by monoclonal B cells, expressing CD20, but negative for CD3−, CD5, CD10, CD138, IgA, IgG, and IgM (Fig. 1d). These cells showed κ−light chain restriction (Fig. 1e, f). Staining with CD138 highlighted less than 1 % plasma cells, while the lymphoma cells do not express CD138. In view of the discrepancy, cholecystectomy was performed showing a plasmacytoma (Fig. 1g). The plasma cells were CD138 positive, CD20 negative, and also positive for cytoplasmic lambda chain and IgA, but negative for IgG, IgM, and κ−light chain restriction. (Fig. 1h, i). They also expressed CD56 and cyclin D1. The double pathology was treated with R-CEOP ×6 (rituximab, cyclophosphamide, epirubicin, vincristine, and prednisolone) together with maintenance with thalidomide and bortezomib (weekly × 16 doses) and radiotherapy to both humerus. A complete remission by radiological and serum immunofixation assessment was shown at 1 year. He refused an autologous stem cell transplantation (SCT). The clonal relationship between the two lesions was investigated by polymerase chain reaction using IgH VJ primers followed by cloning and sequencing, using DNA from the homogeneous plasmacytoma and microdissected lymphoma cells from the trephine biopsy [1]. This showed a direct relationship between the two B cell clones in terms of band size and sequence homology (Fig. 1j and k).

This case is interesting in terms of pathogenesis and treatment. In terms of pathogenesis, cases of concomitant B cell lymphoproliferation and plasma cell dyscrasia are rare and usually of independent clones [2]. The immunophenotyping and light chain expression do not suggest a common evolution from a lymphoplasmacytoid lymphoma. Our case is characterized by clonal homology but discordant light chain expression, immunophenotype, and morphology. Plasma cell myeloma with discordant light chain secretion is well documented [3]. Posttreatment switch in monoclonal protein may also occur after SCT [4]. More dramatically, changes in immunophenotype, morphology, and light chain expression in B cell malignancies after rituximab treatment are also recognized [1, 5, 6]. However, in our case, the clones occurred concomitantly and upfront in distinct cell lineages*.* This may theoretically result from plasma cell differentiation of the malignant B lymphocytes or a divergent evolution from a primordial B cell clone. Pools of malignant clonotypic B cells are known to exist in PCM patients [7]. In terms of treatment, a combination of lymphoma and PCM treatment seems to be effective in controlling both lesions with minimal overlap of toxicity. It is uncertain whether this patient may have survival benefit from an autologous SCT.
